# It takes two to tango: Widening our understanding of the onset of schizophrenia from a neuro-angiogenic perspective

**DOI:** 10.3389/fcell.2022.946706

**Published:** 2022-08-24

**Authors:** Bárbara S. Casas, David Arancibia-Altamirano, Franco Acevedo-La Rosa, Delia Garrido-Jara, Vera Maksaev, Dan Pérez-Monje, Verónica Palma

**Affiliations:** Laboratory of Stem Cells and Developmental Biology, Departamento de Biología, Facultad de Ciencias, Universidad de Chile, Santiago, Chile

**Keywords:** schizophrenia, neurogenesis, angiogenesis, brain development, blood-brain barrier, neurovascular niche, hiPSC

## Abstract

Schizophrenia is a chronic debilitating mental disorder characterized by perturbations in thinking, perception, and behavior, along with brain connectivity deficiencies, neurotransmitter dysfunctions, and loss of gray brain matter. To date, schizophrenia has no cure and pharmacological treatments are only partially efficacious, with about 30% of patients describing little to no improvement after treatment. As in most neurological disorders, the main descriptions of schizophrenia physiopathology have been focused on neural network deficiencies. However, to sustain proper neural activity in the brain, another, no less important network is operating: the vast, complex and fascinating vascular network. Increasing research has characterized schizophrenia as a systemic disease where vascular involvement is important. Several neuro-angiogenic pathway disturbances have been related to schizophrenia. Alterations, ranging from genetic polymorphisms, mRNA, and protein alterations to microRNA and abnormal metabolite processing, have been evaluated in plasma, post-mortem brain, animal models, and patient-derived induced pluripotent stem cell (hiPSC) models. During embryonic brain development, the coordinated formation of blood vessels parallels neuro/gliogenesis and results in the structuration of the neurovascular niche, which brings together physical and molecular signals from both systems conforming to the Blood-Brain barrier. In this review, we offer an upfront perspective on distinctive angiogenic and neurogenic signaling pathways that might be involved in the biological causality of schizophrenia. We analyze the role of pivotal angiogenic-related pathways such as Vascular Endothelial Growth Factor and HIF signaling related to hypoxia and oxidative stress events; classic developmental pathways such as the NOTCH pathway, metabolic pathways such as the mTOR/AKT cascade; emerging neuroinflammation, and neurodegenerative processes such as UPR, and also discuss non-canonic angiogenic/axonal guidance factor signaling. Considering that all of the mentioned above pathways converge at the Blood-Brain barrier, reported neurovascular alterations could have deleterious repercussions on overall brain functioning in schizophrenia.

## 1 Introduction

Schizophrenia (SZ) is a complex psychiatric disorder affecting approximately 1% of the population worldwide (Owen et al., 2016). SZ is characterized by positive (e.g., delusions, hallucinations, psychotic episodes), negative (e.g., anhedonia, reduced speech or movements), and cognitive symptoms (e.g., disorganized speech, cognitive deficits). To date, the treatments for SZ mainly target the positive symptoms, leaving cognitive and negative symptoms undertreated, and are estimated to be efficient for 50–70% of patients, but with important metabolic and neurological side effects (Buckley et al., 2007; Stępnicki et al., 2018).

Despite decades of research, the causes of this disorder remain poorly understood. SZ has been described as a neurodevelopmental disease of multiple etiology, in which both genetic and environmental origins are involved (Susser and St Clair, 2013; Jaaro-Peled and Sawa, 2020). This is supported by the altered expression of development-related genes and the important brain remodeling that occurs around the age of SZ onset, during late adolescence and early adulthood (Kochunov and Hong, 2014; Weinberger, 2017; Rund, 2018).

As in most neurological disorders, the main descriptions of SZ physiopathology have been focused on neuronal deficiencies. Altered cerebral connectivity and brain dynamics, abnormalities in the excitatory/inhibitory balance, as well as failure to specify specific neuron identities have been described (Spencer, 2009; Kantrowitz and Javitt, 2010; Inan et al., 2013; Puvogel et al., 2022). In addition to these neuronal alterations, increasing evidence has linked SZ to vascular impairments. Hypoperfusion in several areas of the brain, reduced cerebral blood flow (CBF), and Blood-Brain Barrier (BBB) dysfunctions have been described (Andreasen et al., 1997; Lopes et al., 2015; Katsel et al., 2017; Baruah and Vasudevan, 2019; Puvogel et al., 2022).

**FIGURE 1:**
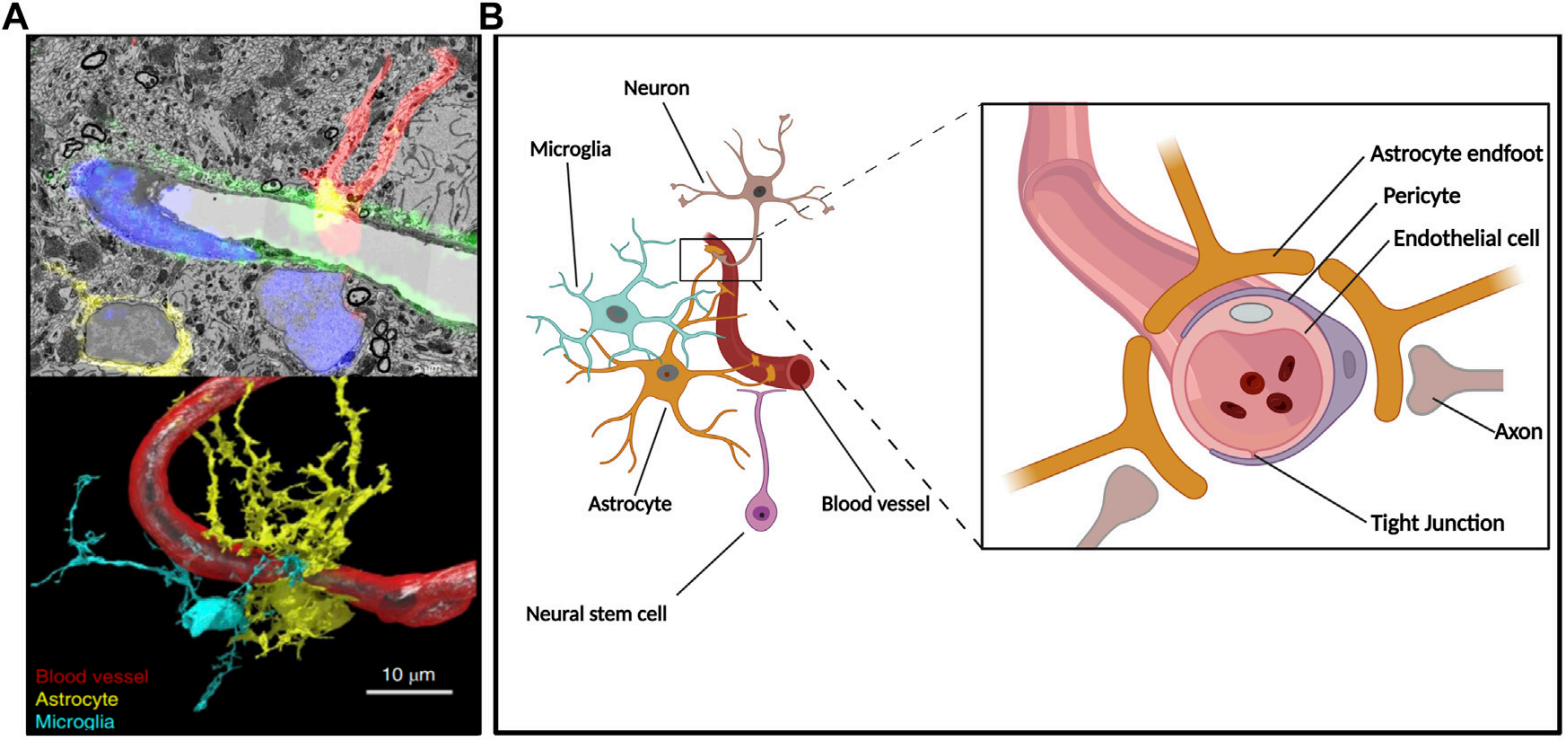
The neuro-angiogenic niche and the NVU. Constitutive components of the NVU reside in a microenvironment where secreted factors, cell-cell interactions, and vascular supply give rise to an interdependent unit. **(A)** Hippocampal NVU comprising blood vessels and surrounding tissue depicted throughout a composition of electron microscopy-acquired sections and its corresponding confocal microscopy fluorescence image (upper panel) as well as subsequent 3D reconstruction (lower panel); reproduced with permission (Fang et al., 2018). **(B)** Simplified diagram depicting components of the BBB at a neurogenic region where radial glia-like NSC are in direct contact with the vasculature.

The human brain critically relies on an elaborate vascular network for its oxygen and nutrient supply (Quaegebeur et al., 2011). This vascular network is the result of the concomitant development of both neural and vascular components in the central nervous system (CNS). Angiogenesis (formation of new blood vessels from pre-existing ones) and vasculogenesis (generation of blood vessels *de novo*) starts early in embryonic development, with the recruitment of angioblasts and endothelial cells (EC) around the newly formed brain constituting the perineural vascular plexus (PNVP) (Bautch and James, 2009; James and Mukouyama, 2011). Then, neural stem cells (NSC) and neural progenitor cells (NPC) will induce the ingrowing of capillaries into the neural tube. Through the action of attractant and repellent molecules acting in gradients and extracellular matrix components, the spatial distribution of vascularization will occur in parallel to the brain’s development and differentiation processes (Engelhardt and Liebner, 2014; Ben-Zvi and Liebner, 2021). This led to the concept of neuro-angiogenesis, which refers to the coordinated development of neurons (neurogenesis) and the formation of new blood vessels (angiogenesis). This communication will be the basis of the induction of BBB characteristics in EC of brain microvessels, as part of a greater module known as the neurovascular unit (NVU) composed of EC, pericytes, astrocytes, and neurons (Figure 1) (Tam and Watts, 2010; Engelhardt and Liebner, 2014; Segarra et al., 2019).

**FIGURE 2:**
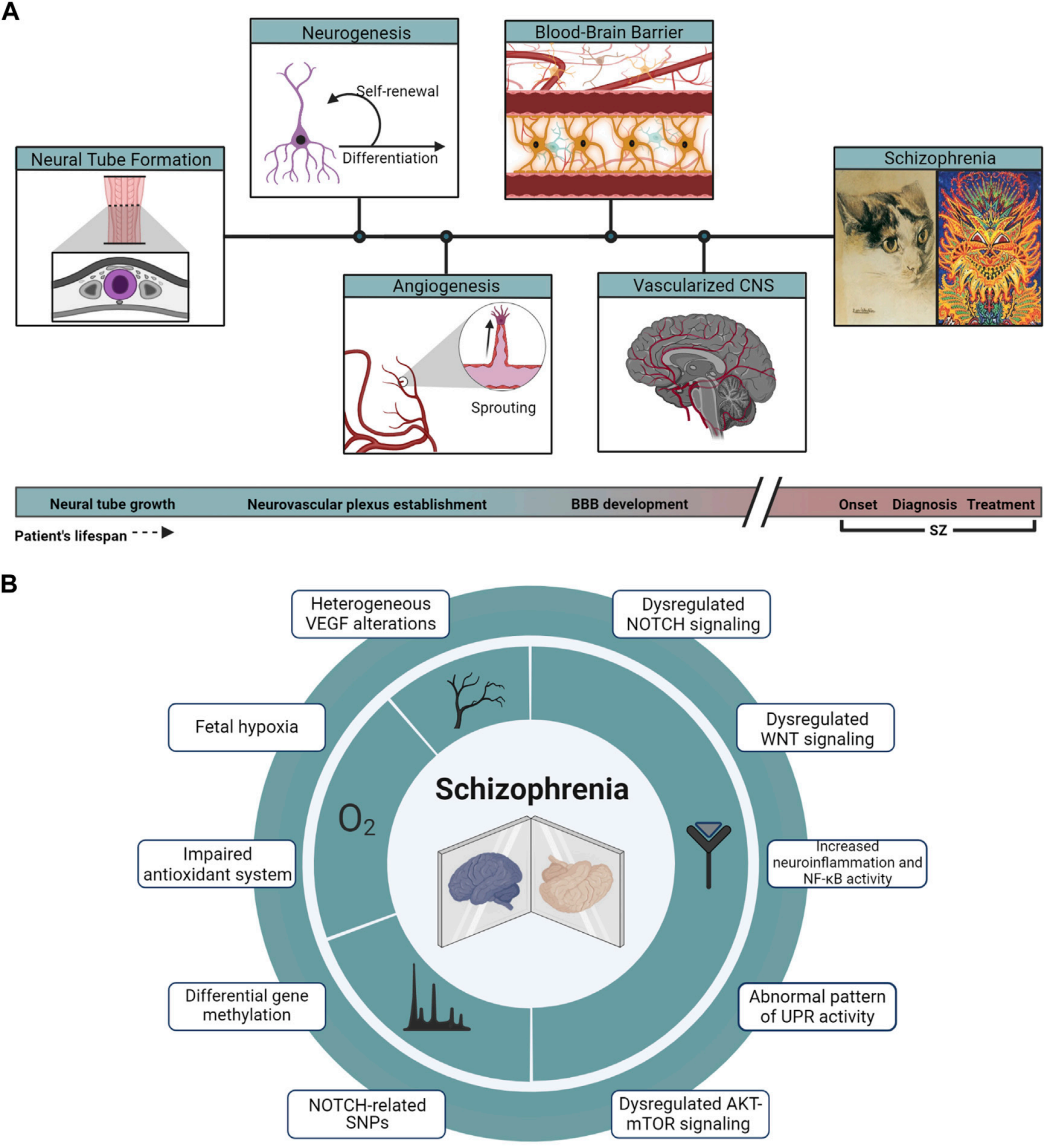
Developmental and molecular hallmarks in SZ. **(A)** From the closure and growth of the neural tube (highlighted in purple from a coronal section across the embryo’s axis) to a vascularized and developed brain, events of neurogenesis and angiogenesis take place across the developing tissue, leading to the establishment of the BBB, a key component in the physiopathology of SZ. Cat illustrations correspond to public domain paintings by Louis Wain, whose changing art style interprets as a representation of the onset and progression of the artist’s SZ. **(B)** Array summarizing the main genomic, signaling, and physiological hallmarks described in the physiopathology of SZ.

The synchronic development of vascular and nervous systems is orchestrated by the molecular crosstalk between both systems. Shared molecular pathways are major players in the communication of the neuronal compartment with the endothelium (Tam and Watts, 2010; Segarra et al., 2019; Peguera et al., 2021). Since neuro-angiogenic signaling plays a crucial role in the adequate structuration of the neurovascular niche, and therefore in brain functioning, SZ can be considered a systemic disease where both nervous and vascular alterations are impacting critical developmental periods (Figure 2A).

In this review, we analyze the alteration of selected signaling pathways and cascades involved in both angiogenic and neurogenic processes and their implications in SZ. We discuss some classical and emerging pathways involved in the pathophysiology of this disease, considering the interconnected network of both vascular and nervous systems as a whole. A better understanding of these processes may lead to the identification of potential biomarkers for the early diagnosis and treatment of SZ.

## 2 SZ is characterized by an impaired antioxidant system: The HIF pathway and oxidative stress

During CNS development, vascularization and subsequent oxygenation are key for changing stem cell behavior from proliferative to differentiative states (Morante-Redolat and Fariñas, 2016). Hypoxia-Inducible Factors (HIFs) constitute a family of heterodimeric transcription factors that regulate oxygen homeostasis, exerting transcriptional control over hundreds of genes in response to changes in oxygen supply. HIFs are also involved in processes such as glycolytic metabolism, proliferation, angiogenesis, and stemness (Semenza, 2014, 2020). Under normoxia, the subunit HIF-1α is actively hydroxylated by an oxygen-dependent mechanism mediated by prolyl hydroxylases (PHDs) and destined for proteasomal degradation. Under hypoxia, such degradation is rescued and HIF-1α translocates into the nucleus where it dimerizes with HIF-1β and regulates gene transcription through its binding to hypoxia response elements (HREs) on target genes (Dengler et al., 2014). In what could appear a counterintuitive observation, PHDs can also be inhibited in hypoxia by reactive oxygen species (ROS) (Gerald et al., 2004). ROS and mitochondrial ROS production can stabilize HIFs in non-hypoxic conditions, as is the case in hyperoxia-mediated induction of neurogenesis or non-hypoxic stabilization of HIF-1α in cerebellar progenitors proliferation via Sonic Hedgehog (SHH)-induced ROS production (Simon, 2006; Hu et al., 2014; Eyrich et al., 2019).

Perturbations in oxygen supply have been long while associated with the physiopathology of SZ and, although there are inconsistencies in independent enzymes or metabolites, there is an overall notion that SZ is characterized by an impaired antioxidant system (J. Q. Wu et al., 2013).

Fetal hypoxia has been described as an environmental risk for SZ and the HIF pathway is considered a crucial target to understand and treat SZ (Tsuang, 2000; Katsel et al., 2017). Association studies show that many of the so-called susceptibility genes for SZ are regulated by hypoxia and/or expressed in the vasculature (Manalo et al., 2005; Schmidt-Kastner et al., 2006; Schmidt-Kastner et al., 2012). In addition, global analyses of the human brain showed that genes associated with metabolism and oxidative stress allow for discrimination of around 90% of SZ patients from healthy subjects, demonstrating the important implication of oxygen regulation in SZ physiopathology (Prabakaran et al., 2004). Given the common genic variation in these genes, the HIF-2 pathway has been proposed as a potential pharmacological target in the treatment of SZ (Reay et al., 2020). Moreover, a hindered or reduced antioxidant capacity was found in some SZ patients (Albayrak et al., 2013; Flatow et al., 2013). Antioxidant markers such as catalase and nitrite exhibited changes during the clinical course of patients, being lower at first-episode psychosis (FEP) and then increased in patients with exacerbation of psychosis and under pharmacological treatment (Flatow et al., 2013). Pharmacological agents commonly used in clinical treatment alter the systemic oxidative status and suggest a potential redox modulation in their therapeutic mechanism of action (Pandya et al., 2013; Pillai et al., 2007; J. Q. Wu et al., 2013; Z. Wu et al., 2012).

Since there is complex crosstalk between oxidative species and HIF pathways, the above-mentioned alterations could rise from a pleiotropic integration of developmental and environmental signals. As such, an increase in ROS by mitochondrial dysfunction (Prabakaran et al., 2004), could trigger events of non-hypoxic HIF stabilization, affecting both development and homeostasis of both vascular and neural lineages, and compromising brain metabolism in SZ. These dysregulations could also affect the course of the illness, for which early targeting of the HIF and ROS pathways could improve patient outcomes.

## 3 Aberrant NOTCH signaling pathway in SZ

The NOTCH pathway is a conserved pathway involved in multiple developmental processes including neural development (Bray, 2016; J. Liu et al., 2010). The essential steps of human NOTCH signaling transduction involve the interaction of ligands with NOTCH receptors. Such an event triggers proteolytic cleavage and subsequent release of the NOTCH intracellular domain (NICD) which is then translocated to the nucleus where it forms a gene activating complex with the transcriptional regulator RBPJ and the co-activator MAML1 (Bray, 2016). There are multiple human ligands and some of them constitute a canonical group defined by the presence of a Delta/Serrate/Lag-2 (DSL) domain and are denoted as Delta-like (DLL) and Jagged (JAG) families, while non-canonical ligands present a high grade of structural diversity and lack the aforementioned DSL domain, such as the well-researched Delta Like Non-Canonical Notch Ligand 1 (DLK1) (D’Souza et al., 2010).

NOTCH signaling is a relevant actor in the vascularization and remodeling of both the embryonic and postnatal brain (Jakobsson et al., 2010; Walchli et al., 2015). Inhibition of NOTCH signaling results in reduced Trans-endothelial Electrical Resistance (TEER), increased permeability, and reduction and delocalization of VE-cadherin and Claudin-5 in brain EC (Derada Troletti et al., 2018). As neural counterparts, Notch2 and 1 have shown to control quiescence and differentiation of ventricular–subventricular zone (V-SVZ) quiescent and activated NSC, respectively, in crosstalk involving the HIF pathway (Basak et al., 2012; Engler et al., 2018).

Early evidence associating NOTCH signaling and SZ came from linkage mapping reports and association studies through genotyping of blood samples. The *NOTCH4* gene at chromosome 6p has been described as a potential susceptibility gene with multiple SNPs that suggest the presence of several SZ-associated variants (S. Wang et al., 1995; Wei and Hemmings, 2000; X. Zhang et al., 2004). Further research and integration of genome-wide association studies (GWAS) in SZ have associated more variants of NOTCH pathway components, such as *NOTCH4*, *NUMBL,* and *FURIN*, describing the latter as a pleiotropic SNP also involved in major depressive disorder and bipolar disorder (Passos Gregorio et al., 2006; H. Wang et al., 2022; X. Yang et al., 2013; B. Zhang et al., 2015). In addition to the increased variants of NOTCH pathway genes in SZ, epigenetic analysis has revealed evidence of differentially methylated regions of NOTCH pathway genes in SZ patients (Shen et al., 2021).

Regarding the expression of pathway genes, it has been reported that the levels of Notch ligands DLL2 and DLK1 were higher in plasma from SZ patients, along with multiple other components of the pathway that were also differentially regulated in whole blood samples relative to healthy subjects (Hoseth et al., 2018b). The increase in the plasmatic levels of NOTCH pathway ligands is suggested to promote an attenuation of NOTCH signaling; since DLK1 is a non-canonical ligand with inhibitory effects via NOTCH1. The interpretation of the effects of an increment in plasmatic levels of DLL1 is more diffuse as the cleavage of DLL1 could relieve cis-inhibitory effects over NOTCH receptors while also yielding C-terminal fragments that compete with NOTCH receptors for proteolytic machinery and thus inhibit signaling as a result of impaired cleavage processing of receptors (Mishra-Gorur et al., 2002; LaVoie and Selkoe, 2003; Falix et al., 2012). *In vitro* modeling of SZ utilizing human-induced pluripotent stem cells (hiPSC)-derived neurons, which represent early stages of brain development, showed a decreased expression of NOTCH pathway components such as *NOTCH1*, *HEY2* or *DLL1* when compared to healthy samples (Brennand et al., 2011).

Interestingly, while SZ patients presented reduced plasma levels of the transcriptional regulator RBPJ, its levels were significantly increased in SZ patients undergoing lithium treatment (Hoseth et al., 2018b). In an MK-801-induced murine model of SZ, the antipsychotic risperidone enhanced the Notch pathway activity and rescued cognitive deficits, effects that were abrogated by *Notch1* knockdown (Xue et al., 2017). In human NT2-differentiated neurons, other neuroleptics for SZ treatment (e.g., amisulpride, aripiprazole, and clozapine) decreased the expression of NOTCH signaling components (Panizzutti et al., 2021), for which we could be observing a contribution of treatment along with the disease progression in the expression of those genes. Therefore, the expression of NOTCH pathway genes can vary during the time course of the life of patients and may be related to epigenetic control.

Due to the increased levels of NOTCH ligand JAG1 found in the internal capsule of SZ *postmortem* brains, and its negative correlation with the expression of genes related to oligodendrocyte function, it has also been proposed that oligodendrocyte and myelin development is hindered in SZ (Kerns et al., 2010). Cuprizone intoxication has been used to establish a murine model with oligodendrogenic aberrations, white matter lesions, and behavioral changes that could mimic SZ (Xu et al., 2009; Chandran et al., 2012). In this model of demyelination, Notch components, such as Notch1 and Hes1/5 were reduced, and amelioration of cuprizone effects by the antipsychotic quetiapine was found to be Notch-dependent (H. N. Wang et al., 2015).

In summary, the Notch pathway is importantly implicated in SZ for including several high polygenic risk genes. Since this pathway also has an important role in angiogenesis and BBB function, it would be important to evaluate the effect of NOTCH pathway dysregulation in those processes, especially given the impact of SZ treatment on the pathway component expression.

## 4 Vascular endothelial growth factor (VEGF) signaling is highly heterogeneous between SZ patients

Vascular endothelial growth factors (VEGFs) are essential proangiogenic factors that comprise seven members: VEGFA, VEGFB, VEGFC, VEGFD, VEGFE, VEGFF, and PlGF (Hoeben et al., 2004). VEGFs exert numerous functions through their high-affinity binding to receptor tyrosine kinases VEGFR-1, -2, and -3 and co-receptors neuropilin-1 (NRP1), neuropilin-2 (NRP2), and heparan sulfate proteoglycans (HSPGs) (Simons et al., 2016). During development, CNS angiogenesis is largely controlled by neural-derived VEGF, which stimulates PNVP formation and blood vessel sprouting from the PNVP into the CNS parenchyma. Furthermore, VEGF expression in developing brain EC is key for neurogenesis (Paredes et al., 2018). In the adult brain, VEGF also participates in neurogenesis, neuroprotection, and synaptic plasticity (Lopes et al., 2015), and has an important role in regulating vessel permeability and matching microvascular density to perfusion demands (Licht and Keshet, 2013).

A great sum of evidence relates alterations in VEGF signaling with SZ. Analysis of data obtained from the Common Mind Consortium (CMC) study has determined that VEGFA is among the genes with the greatest variability among SZ patients, compared to healthy subjects (Huang et al., 2020). But reports of VEGFA levels in SZ are rather contradictory. The first evidence pointing to alterations of VEGFA expression in SZ, described lower mRNA levels in the dorsolateral prefrontal cortex of *postmortem* SZ brains (Fulzele and Pillai, 2009). Also in *postmortem* studies, it has been found that VEGFR-2 protein levels in the prefrontal cortex are diminished in SZ in comparison with controls (Howell et al., 2011; Hino et al., 2016). This deficiency in VEGFA signaling has also been corroborated by *in vitro* SZ hiPSC modeling using NSC. Interestingly, brain microvascular EC derived from SZ-hiPSC have a decreased angiogenic response when stimulated with VEGFA (Casas et al., 2018; Casas et al., 2022). However, VEGF protein levels in the superior temporal gyrus (STG) are not significantly different between SZ and control groups (Izumi et al., 2021).

Several studies have found significantly lower plasmatic VEGFA levels in SZ patients when compared to healthy controls (Lee et al., 2015; Xiao et al., 2018; Ye et al., 2018). Nevertheless, a meta-analysis revealed no differences in VEGF blood levels between drug-naïve first-episode SZ patients and controls. Nevertheless, when the analysis was restricted to high-quality studies only, significantly increased VEGF levels were observed in these patients compared to controls (Çakici et al., 2020).

Despite basal serum levels of VEGFA, an increase in VEGFA after treatment of SZ has been reported (Pillai et al., 2016; Balõtšev et al., 2017; Frydecka et al., 2018; Misiak et al., 2018; Ye et al., 2018; Xiao et al., 2019). Moreover, higher VEGFA basal levels could predict positive treatment response and VEGFA levels decrease with the severity of illness and cognitive impairment in SZ (Xiao et al., 2018; Zhao et al., 2019). Conversely, another study found that VEGF serum levels of drug-naïve FEP patients decreased after they completed 7 months of antipsychotic treatment (Haring et al., 2015).

In conclusion, heterogeneous VEGF alterations may exist between SZ patients. Given the importance of VEGF signaling in neurovascular processes, its dysregulation could contribute to the pathophysiology of SZ, characterized by microvascular anomalies and impaired angiogenesis. Moreover, the cognitive dysfunction associated with the disease could be related to aberrant VEGF signaling impacting neurodevelopment and neural plasticity (Howell and Armstrong, 2017).

## 5 Dysregulation of both canonical and non-canonical WNT signaling in SZ

The canonical WNT signaling pathway is a key regulator of a large number of biological processes and, as expected, its alteration is associated with many human diseases. This signaling pathway is characterized by the activation of gene expression regulated by β-catenin. WNT proteins bind to transmembrane receptors of Frizzled (FZD) family and co-receptors Low-density lipoprotein receptor-related protein (LRP)5/6, which leads to the disassembly of the β-catenin destruction complex and prevents β-catenin proteasomal degradation. Therefore, the stabilized β-catenin accumulates in the cytoplasm and is subsequently translocated into the nucleus to form a complex with LEF/TCF proteins and regulates the expression of WNT target genes. The WNT signaling pathway is critical in processes of neural development as well as adult neurogenesis, synaptic transmission, and plasticity (Mulligan and Cheyette, 2016; Varela-Nallar and Inestrosa, 2013; K. Yang et al., 2016).

Canonical WNT signaling pathway is activated in CNS blood vessels during development and is crucial for BBB formation and the expression of many specific influx transporters, such as GLUT-1. During this process, NPC expresses WNT ligands in a regionally specific manner, while CNS EC express WNT receptors (Stenman et al., 2008; Daneman et al., 2009). This signaling is also necessary for BBB maturation and function *in vivo*. In mice, deletion of β-catenin specifically in EC at stages of BBB maturation is associated with BBB disruption (Liebner et al., 2008). There is also evidence showing that activation of this pathway supports the maintenance of BBB properties in adulthood, mainly through the secretion of WNT ligands by astrocytes (Blanchette and Daneman, 2015; Guérit et al., 2021).

Multiple studies suggest that SZ is associated with an altered canonical WNT signaling pathway. The disease has been associated with genetic variants in several pathway-related genes, including *TCF4*, *CTNNB1*, *CHD8*, *DKK1*, *DKK4,* and *KREMEN1* (Mulligan and Cheyette, 2016).

SZ patients have lower plasma levels of WNT inhibitors DKK1 and sclerostin (SOST) (Hoseth et al., 2018a). Furthermore, whole blood samples of SZ patients show reduced mRNA expression of several WNT pathway genes; pointing to an attenuated canonical WNT signaling in SZ (Kalkman, 2009; Hoseth et al., 2018a). *In vitro* models seem to support this notion. Six-week-old neurons obtained from SZ hiPSC show alterations in transcript levels of many WNT pathway genes, such as *AXIN2*, *WNT2B*, *WNT3*, *TCF4*, *LEF1*, *LRP5,* and *WNT7A*. The same authors showed that the decreased expression of *WNT7A* in SZ neurons can be reversed with 3 weeks of treatment with the antipsychotic Loxapine, which also improved neuronal connectivity *in vitro* (Brennand et al., 2011). Also, mRNA expression of secreted WNT inhibitors DKK1, DKK2, SFRP2, and SFRP4 is increased in SZ hiPSC-derived NPC (Topol et al., 2015).

In addition to the canonical WNT/β-catenin pathway, the WNT ligands can activate signaling cascades without interaction with β-catenin, so-called, non-canonical pathways (Montcouquiol et al., 2006). Analysis of SZ patient blood samples show enrichment of non-canonical WNT signaling components (FZD7 and NFAT) (Hoseth et al., 2018a).

In conclusion, the evidence presented highlights a dysregulation of both canonical and non-canonical WNT signaling in SZ, with an important hypofunction of the canonical pathway. This could have profound consequences on BBB structure and function, as well as neural development and connectivity.

## 6 AKT/mTOR defective signaling suggests systemic alterations in SZ

The AKT/mTOR signaling pathway regulates multiple cellular functions such as nutrient uptake, cell proliferation, growth, autophagy, apoptosis, and migration (L. Wang et al., 2017). This pathway has numerous components and interactors, including mTORC1, one of AKT’s main downstream effectors (Rosen and She, 2006; Efeyan and Sabatini, 2010; Zoncu et al., 2011). The AKT/mTOR pathway is activated in response to inputs from regulatory molecules like growth factors, ATP, nutrient concentrations, energy, and ambient oxygen levels. Once activated, it modulates protein homeostasis via the phosphorylation of the S6K1 kinase, leading to cell proliferation and growth (Ma and Blenis, 2009; Bar-Peled et al., 2013; Ben-Sahra et al., 2013; Koehl et al., 2021).

AKT has been related to EC migration and angiogenesis via the promotion of increased VEGF secretion, due to the upregulation of HIF-1α (Manning and Cantley, 2007; Abeyrathna and Su, 2015). The AKT/mTOR pathway acts upon and influences the cell fate lineage of NSC, thus playing a crucial role in neuronal shape and size, dendritic arborization, spine morphology, axon outgrowth, and synaptic plasticity (Götz and Huttner, 2005; Tavazoie et al., 2005). It is also implicated in numerous neurological processes such as learning, memory, and feeding (Bockaert and Marin, 2015; Garza-Lombó and Gonsebatt, 2016). mTORC1 impairments have been implicated in brain development defects like defective synaptogenesis or connectivity and in pathogenic conditions such as epilepsy, autism, intellectual disability, dementia, traumatic brain injury, brain tumors, and ischemic injuries (Lipton and Sahin, 2014; Bockaert and Marin, 2015; Huber et al., 2015; Licausi and Hartman, 2018).

Recent reports point to the downregulation of the AKT/mTOR signaling cascade in SZ *postmortem* brains where a decreased protein expression and activity of AKT and mTOR kinases was found (Chadha and Meador-Woodruff, 2020). Interestingly, while downstream S6RP phosphorylation is reduced, downstream of mTOR complex II, PKCα phosphorylation is increased in SZ *postmortem* brains (Chadha et al., 2021). Furthermore, rats treated chronically with the antipsychotic haloperidol had increased levels of S6RP phosphorylation (Chadha et al., 2021). GSK-3ß, a major target of AKT, was also shown to be reduced at a transcriptional, protein, and phosphorylation level in *postmortem* frontal cortex and peripheral lymphocytes of SZ human patients (Kozlovsky et al., 2004; McGuire et al., 2017; Ibarra-Lecue et al., 2020). In addition to the clinical and *postmortem* data, *ex vivo* approaches using protein and mRNA data from patient-derived olfactory neurospheres have highlighted the mTOR signaling pathway as a significant dysregulated pathway in SZ (English et al., 2015).

Overall, the findings point to an important dysregulation in the AKT-mTOR signaling pathway in the SZ brain, which could lead to important functional consequences.

## 7 Non-canonical angiogenic signaling cues are compromised in SZ

Besides having morphological and structural similarities, with an emphasis on axonal growth cones and EC tip pathfinding, vessels and nerves share several signaling cascades. Numerous factors that were initially described as axonal guidance molecules also exert an important angiogenic function. Among these factors, we found: Netrins, Semaphorins, Ephrins, and Slits (Wälchli et al., 2015). Many also have an important role in the formation and maintenance of BBB (Blanchette and Daneman, 2015). Therefore, alterations in neurovascular communication could contribute to the development of SZ.

Netrins are secreted proteins, that regulate many cellular processes during brain development such as cell migration, adhesion, and proliferation; as well as pre and postnatal angiogenesis (Yamagishi et al., 2021). Netrin-1 (NTN1), the main netrin in CNS, can exert attractive/positive or repulsive/negative responses depending on the concentration and presence of different receptors in the target cell. Notably, NTN1 can regulate BBB function by decreasing permeability through the induction of tight junction protein expression (Podjaski et al., 2015).

Genomic studies have found an association of polymorphisms in the Deleted Colorectal Cancer gene (*DCC*), one of the main NTN1 receptors in the CNS, in SZ (Grant et al., 2012; Z. Wang et al., 2018). These polymorphisms could alter DCC protein expression affecting NTN1 signaling (Vosberg et al., 2020). Polymorphisms of another NTN1 receptor, UNC5B, have also been correlated with SZ (J. Tang et al., 2019). Interestingly, membrane-bound Netrins, NTN-G1 and NTN-G2, were downregulated in the SZ temporal lobe; these proteins are important for neural development and synaptic activity but have not been implicated in angiogenesis so far (J. Tang et al., 2019). *In vitro* experiments using hiPSC-derived NSC and neurons have shown decreased expression of NTN1 in SZ-derived cells, compared to healthy control (Brennand et al., 2011; Casas et al., 2018).

Semaphorins are a large protein family of molecules that regulate axonal guidance and synapse formation during development (Carulli et al., 2021). They also participate in other functions including immune regulation, extracellular matrix remodeling, and angiogenesis. Canonically semaphorins have been described as chemorepellents, but some of them can also exert attractive effects (such as SEMA3D, SEMA3E, and SEMAF), an important cue for neurovascular communication (Iragavarapu-Charyulu et al., 2020). For instance, semaphorin 3G (SEMA3G) secreted from the endothelium, activates neuronal receptors neuropilin-2/PlexinA4 regulating synaptic structure and function; SEMA4D induces BBB dysfunction by the disruption of endothelial tight junctions, glial activation, neuronal collapse and inhibition of oligodendrocyte differentiation. Neuron-derived SEMA3A can induce vascular permeability and has been proposed as a contributor to BBB disruption and brain damage (Cerani et al., 2013; Hou et al., 2015; Smith et al., 2015; Tan et al., 2019; M. Yang et al., 2019).

Analysis of *postmortem* brain shows that *SEMA3A*, *SEMA4D,* and *SEMA6C* as well as semaphorin receptor *PlexinB1* are upregulated in SZ (Gilabert-Juan et al., 2015). The upregulation of SEMA3A has been also evidenced in the SZ cerebellum of adult subjects and by the *in vitro* modeling of SZ-hiPSC-derived NSC (Eastwood et al., 2003; Casas et al., 2018).

The Slit family comprises 3 proteins (SLIT1, SLIT2, and SLIT3) that interact with ROBO to regulate several physiologic processes during development (Tong et al., 2019). The SLIT/ROBO pathway plays an important role in neural progenitor proliferation and migration, maturation of neocortical neurons, and axonal guidance (Gonda et al., 2020). Slit2 overexpression has been proved to increase vessel density in transgenic mice. Interaction with Robo1 also reduces cell-cell adhesion of EC increasing permeability (Han and Geng, 2011). Of note, the administration of recombinant Slit2 reduced BBB disruption due to brain surgical injury (Han and Geng, 2011). Therefore, it is proposed that the Slit/ROBO pathway has a regulatory function in angiogenesis and BBB.

GWAS performed on peripheral blood cells of patients with SZ has identified *SLIT1*, *SLIT3*, and *ROBO1* as susceptibility genes for this disease. Moreover, an SNP in one locus of *ROBO2* significantly correlates with SZ (Potkin et al., 2009; Z. Wang et al., 2018).

Ephrins and their receptors Eph are cell membrane proteins known to mediate cell-cell communication, regulate adhesion, neurogenesis, neuron migration, and axonal guidance (Laussu et al., 2014). The ephrin/Eph signaling pathways play an important role in vascular system development, regulating EC differentiation, angiogenesis, and also BBB formation and maintenance (Malik and Di Benedetto, 2018). During early vasculogenesis, VEGFA-induced Ephrin A signaling by receptor EphA2 increases angiogenesis, later on, the inhibition of EphA2 is necessary for tight junction protein expression and proper BBB formation (Zhou et al., 2011; Malik and Di Benedetto, 2018).

There is little genomic and clinical data available regarding this pathway in SZ. One study found a significant association between SNPs in the *EhprinB2* gene and SZ (R. Zhang et al., 2010). *In vitro* modeling of SZ-hiPSC-derived NSC, neurons, and astrocytes has provided more information, describing alterations in the expression of Eprhin A1, A5 and B1 (Brennand et al., 2011; Casas et al., 2018; Koskuvi et al., 2020).

All the above-cited reports are in line with current knowledge of important impairments of neuronal function in SZ. The studies describe alterations in the expression of secreted molecules for which the analysis of this data, from a new perspective that focuses on the neurovascular niche, could determine the impact of signaling alterations in these pathways on the overall functioning of BBB and neurovascular coupling.

## 8 Nuclear factor-kappa B (NF-κB) and inflammation in SZ

Nuclear factor-kappa B (NF-κB) transcription factors regulate the expression of numerous genes involved in pleiotropic functions, ranging from immune and inflammatory responses, apoptosis, cell proliferation, differentiation, and survival (Oeckinghaus and Ghosh, 2009). The NF-κB family consists of 5 members including RelA (p65), RelB, c-Rel, NF-κB1 (p50/p105) and NF-κB2 (p52/p100), that form different homo- or heterodimers (Chen and Greene, 2004). The canonical pathway is induced by microbial products and proinflammatory cytokines such as tumor necrosis factor α (TNFα) and interleukin-1 (IL-1), while the non-canonical pathway is activated by certain members of the TNF family (Lymphotoxin β, CD40 ligand, B cell activation factor) (Lawrence, 2009; Oeckinghaus and Ghosh, 2009). In turn, NF-κB plays a role in the expression of other proinflammatory genes including cytokines, chemokines, and adhesion molecules (T. Liu et al., 2017).

NF-κB signaling is well known as a regulator of both embryonic and adult neurogenesis (Bortolotto et al., 2014; Y. Zhang and Hu, 2012). Indeed, it has been reported that this signaling pathway is implicated in different processes of neurogenesis, including NSC/NPC proliferation and apoptosis, differentiation, migration of neuroblasts, maturation and plasticity of nascent neurons (Y. Zhang and Hu, 2012). Cytokines, including IL-1β and TNFα, influence neurotransmission by altering the metabolism of neurotransmitters and their release (Dunn et al., 1999). Several studies that have implicated NF-κB in EC activation and angiogenesis, especially in the context of cancer (Tabruyn and Griffioen, 2008). There is also evidence that dysregulation of NF-κB target genes may lead to impaired angiogenesis and reduced brain EC migration (Zhuang et al., 2017). Since inflammatory mediators have a role during brain development, several studies have investigated their involvement in neuropsychiatric disorders (Jiang et al., 2018).

There is considerable evidence showing increased levels of proinflammatory molecules in the brain, blood, and cerebrospinal fluid of SZ patients (Fillman et al., 2013; Müller, 2018). Patients with higher plasma cytokine levels, named “high inflammation” biotype, exhibit higher cognitive deficits and brain volume reductions (Fillman et al., 2016). Higher mRNA levels for NF-κB family members RELA and c-REL, as well as for multiple receptors from both the canonical and non-canonical pathways, and their respective kinases have also been observed in SZ *postmortem* brain (Volk et al., 2019). Consistent with these reports, SZ patients exhibit increased serum levels and peripheral blood mononuclear cell (PBMC) mRNA expression of IL-1β and TNFα. Furthermore, NF-κB activity is higher in patients’ PBMCs than those in healthy subjects and is positively correlated with a higher mRNA expression of cytokines (Song et al., 2009). Recently, it was reported that some brain EC markers, such as intercellular adhesion molecule-1 (ICAM1) and VE-cadherin, are dysregulated in the SZ brain both in “high inflammation” and “low inflammation” subgroups (Cai et al., 2020). This endothelial altered profile is replicated *in vitro* when incubating human brain EC with IL-1β, as *ICAM1* mRNA was up-regulated in a dose-dependent manner (Cai et al., 2020), which corroborates an important detriment of endothelial function in “high inflammation” SZ patients.

These results suggest that SZ is associated with elevated pro-inflammatory cytokines in both blood and brain and, in turn, increased NF-κB activity, constituting a positive feedback loop. This may contribute to the pathogenesis of SZ through involvement in both neurogenesis and angiogenesis during development, compromising processes such as neurotransmitter release, cell proliferation, differentiation and migration, and BBB integrity.

## 9 Unfolded protein response (UPR) is disturbed in SZ

The accumulation of unfolded proteins in the endoplasmic reticulum (ER) induces the unfolded protein response (UPR), an evolutionarily conserved group of signal transduction pathways to sense and respond to ER stress (Bernales et al., 2006; Lin et al., 2008). Three major ER transmembrane stress sensors trigger the UPR signaling pathway: protein kinase RNA-like ER kinase (PERK), activating transcription factor 6 (ATF6), and inositol-requiring enzyme 1 (IRE1) (Ron and Walter, 2007). Under normal conditions, these stress transducers are in an inactive state by binding with the ER chaperone BiP (Jain, 2017). Under stress, BiP dissociates from PERK, ATF6, and IRE1, allowing UPR sensors to be activated (Malhotra and Kaufman, 2007). Each of these three signaling branches involves the activity of multiple downstream molecules, such as transcription factor X-box binding protein 1 (XBP1) or eukaryotic translation initiation factor 2α (eIF2α), all of which aim to restore ER homeostasis by increasing the expression of genes encoding chaperones and proteins associated with apoptosis, and by attenuating protein synthesis (Jain, 2017).

Although the UPR is commonly associated with increased cellular stress and misfolded proteins, it is required in numerous cellular functions (Hetz, 2012). For example, the UPR plays a role in the proliferation, differentiation, maturation, and viability of CNS cells, including neurons, NSC, and glial cells. UPR signaling is a mechanism that controls neurogenesis and brain development, as well as NSC self-renewal and astrogenesis (Murao and Nishitoh, 2017). Furthermore, in the context of angiogenesis, the classical pathways of the UPR have been described to have modulatory properties. Several studies suggest a role for UPR in embryonic vascularization and maintenance and survival of EC, as well as in the regulation of pro-and anti-angiogenic modulators, both in physiological and pathological contexts (Binet and Sapieha, 2015). Since cells with a high secretory load, such as neurons and glial cells, are susceptible to ER stress, fine-tuning of UPR signaling is required. There is increased sensitivity of the brain to abnormalities in the UPR, which is evidenced by its implication in many neurodegenerative diseases and psychiatric disorders, including SZ (Scheper and Hoozemans, 2015; Kim et al., 2021).

Recent reports show that the UPR is dysregulated in the dorsolateral prefrontal cortex of patients with SZ (Kim et al., 2021). The authors observed increased protein expression of BiP and decreased p-IRE1α, the activated state of IRE1α. They also showed decreased p-JNK2 and increased sXBP1, both downstream targets of the IRE1 arm of the UPR (Kim et al., 2021). These findings are consistent with those obtained through proteomic studies, where BiP is dysregulated within the corpus callosum and the dorsolateral prefrontal cortex of SZ patients, suggesting the involvement of cellular stress in this psychiatric disorder (Sivagnanasundaram et al., 2007; Martins-de-Souza et al., 2009). Furthermore, as described above, the UPR is important for neural development, especially the IRE1-XBP1 pathway, as its dysregulation compromises the proper trafficking of glutamate receptors and expression of GABAergic markers during development (Shim et al., 2004; Hayashi et al., 2008). This is consistent with previous reports demonstrating protein abnormalities and may explain the dysregulation of neurotransmitter systems described in SZ (Nucifora et al., 2019; McCutcheon et al., 2020).

Altogether, these results suggest an abnormal pattern of UPR activity in SZ, specifically on the IRE1 axis, and consequent alterations in protein processing. Although evidence indicates the possible involvement of UPR dysregulation in neurodevelopment, there may also be impairment of endothelial function, a matter which should be further investigated.

## 10 The role of MicroRNAs (miRNAs) in SZ: The cases of miR-137 and miR-19

miRNAs, as pleiotropic post-transcriptional regulators of gene expression, have gathered interest for both their potential as biomarkers of SZ and as tools to provide more complex mechanistic and molecular explanations to the diffuse array of altered gene expression underlying SZ (Zhu et al., 2009; He et al., 2018). Overall miR-137 regulates multiple genes associated with processes such as proliferation, stemness, angiogenesis, barrier permeability, or chromatin remodeling (Y. Wang et al., 2020). miR-137 regulates the balance between proliferation and differentiation of NSC and forms a regulatory loop with transcription factors such as SOX2 (Boyer et al., 2005; Smrt et al., 2010; Szulwach et al., 2010; Channakkar et al., 2020). In an *in vitro* model of Brain Tumor Barrier, the genetic manipulation of miR-137 alters barrier permeability and expression of key actors like Occludin, ZO-1, ZO-2, and FOXC2, the latter two being a direct target of miR-137 (Yu et al., 2017).

The Psychiatric GWAS Consortium has reported a strong association between the SNP rs1625579 of miR137 and SZ (Ripke et al., 2011). Moreover, miR-137 is upregulated in SZ and several variants have been associated with its physiopathology (Lett et al., 2013; Chen et al., 2021). The SNP rs1625579 in miR-137 can be a predictor for earlier age of onset of SZ and correlates with severity of patient symptoms (PANSS score) (D. Zhang et al., 2021). The 4-repeats variable number tandem repeat variant rs58335419 (VNTR_4_) has been associated with altered cortical morphology and severe cognitive deficit in SZ patients (Mahmoudi et al., 2021).

Animal models have correlated the expression of the miR-137 variant with an alteration in hippocampus-dependent learning, long-term potentiation, and expression of presynaptic proteins (Siegert et al., 2015), all phenotypes that have been described in SZ.

In addition to its direct implication in SZ, it seems that miR-137 can regulate signaling pathways that are commonly altered in SZ patients. It has been reported that *Notch1* is a target of miR-137, which acts as a regulator of Notch signaling in neurons. Consistent observations in human adipose cells have shown that miR-137 binds directly to the *NOTCH1* 3′UTR region and ultimately positively regulates *HES1* (Shi et al., 2017; Fan et al., 2021). Recent evidence provides insight into the role of miR-137 in response to oxidative damage and neuroprotection with a proposed neuroprotector and anti-inflammatory role against chemical agents and artery occlusion (Y. Tang et al., 2021; Tian et al., 2020). High levels of miR-137 are associated with mitochondrial dysfunction in SZ patients, shedding light on a mechanistic model, linking impairment of cortical parvalbumin interneuron, oxidative damage, and altered activity rhythms (Khadimallah et al., 2021).

The involvement of miR-137 in oxidative stress and the NOTCH pathway presents an interesting direction for future research given the relevant role of oxygenation in SZ when understood as a developmental and neurovascular disorder.

Another brain-specific miRNA dysregulated in SZ is miR-19, which is involved in NSC proliferation and promotes neural differentiation and migration. Recently, miR-19 has been indicated as a major hub for abnormal cellular phenotypes manifested in SZ (Balan et al., 2019). Interestingly, miR-19b, but not miR-19a, is elevated in peripheral blood of SZ patients (Horai et al., 2020).

Overall, these findings support the potential for neurodevelopmental-related miRNAs to be used as indicators for SZ.

## 11 Discussion

SZ is a complex multifactorial disease imparted by the polygenicity and interactions with environmental factors. For achieving an integral understanding of this mental illness, is important not to only focus on the actual state of patients, but to be able to follow its origin, onset, and visualize its outcome. From a neurodevelopmental perspective, SZ traces its origins to the early stages of brain formation, where developmental trajectories will be affected generating predisposition to SZ onset. From FEP and diagnosis, the trajectories of patients living with SZ may also take different paths: remission, mild to severe symptomatology, resistance to treatment, and so on. In all this complexity, we must keep in mind that the brain is composed of an intricate network of vessels that influences brain formation from embryogenesis to adulthood (Figure 2A).

In this review, we emphasize the importance of acknowledging the presence of this complex and dynamic neuro-angiogenic niche for the study of SZ, since both neural and vascular components are constantly interacting with each other and share several signaling pathways (Table 1). The pathways reviewed in this article have a reported function both in the nervous and endothelial components of the neurovascular niche, with reported alterations in SZ that could have deleterious repercussions on overall brain functioning.

**TABLE 1:** Major molecular participants underlying neurodevelopmental processes of angiogenesis, BBB formation, and neurogenesis.

*Process*	Key signaling molecules/pathways	References
*Angiogenesis*	- Hypoxia-Inducible Factors (HIFs)	(Semenza, 2014, 2020; Abeyrathna and Su, 2015)
	- VEGF/NOTCH signaling	(Jakobsson et al., 2010; Walchli et al., 2015; Paredes et al., 2018)
	- WNT/ß-catenin signaling	(Martowicz et al., 2019)
	- Non-canonical WNT ligands	(Korn et al., 2014)
	- Unfolded Protein Response (UPR) signaling	Zhuang et al. (2017)
	- Nuclear Factor-Kappa B (NF-kB)	(Tabruyn and Griffioen, 2008; Binet and Sapieha, 2015)
*BBB formation*	- NOTCH signaling pathway	Derada Troletti et al. (2018)
	- Canonical WNT signaling pathway	(Liebner et al., 2008; Blanchette and Daneman, 2015; Guérit et al., 2021)
	- Nuclear Factor-Kappa B (NF-kB)	Volk et al. (2019)
*Neurogenesis*	- WNT signaling pathway	(Coullery et al., 2016; Arredondo et al., 2020)
	- AKT/mTOR pathway	(Götz and Huttner, 2005; Abe et al., 2010; Polchi et al., 2018)
	- Nuclear Factor-Kappa B (NF-kB)	(Y. Zhang and Hu, 2012)
	- Unfolded Protein Response (UPR) signaling	(Shim et al., 2004; Hayashi et al., 2008; Murao and Nishitoh, 2017)

As reported previously, SZ has an important and highly variable genetic component where more than 100 loci have been significantly associated with SZ (Ripke et al., 2011; Ripke et al., 2014). From a genetic perspective, several pathways analyzed in this review present genetic variants associated with SZ. Genes from the HIF, NOTCH, WNT, and axonal guidance pathways (NTN, ROBO, and Ephrin), present genetic variants, such as SNPs, that have been significantly associated with SZ. From these pathways, the NOTCH and WNT pathways are described as having variants in several of their pathway components, increasing the risk of developing the disease. In addition to their well-known role in neurodevelopment, these pathways are important regulators of angiogenesis, a process that is proposed to be downregulated in SZ patients (Lopes et al., 2015; Katsel et al., 2017; Casas et al., 2018; Casas et al., 2022).

Evaluating the course of illness, various studies report significant variations in analytes when analyzed at different time points of the disease, as FEP, antipsychotic treatment, and treatment resistance. In this context, antioxidants, members of the NOTCH pathway, and VEGFA exhibit changes after treatment. For the antioxidant molecules and VEGFA, these variations should not be associated with drug mechanisms but rather with the inherent characteristics of patients. In this line, it has been proposed that plasmatic levels of VEGFA, which is reported as one of the most variable expressed genes among SZ patients, may be a predictor of patient outcome. On the other hand, the expression of RBPJ, a transcriptional regulator of the NOTCH pathway, which was found to be reduced in patient plasma, is increased after treatment. Animal models and *in vitro* studies indicate that this increment in RBPJ expression may be a direct consequence of an antipsychotic treatment. A similar observation has been reported for the phosphorylation of S6RP, a downstream mediator of the mTOR/AKT pathway, which is reduced in the SZ *postmortem* brain. Long treatments of the antipsychotic haloperidol increase S6RP phosphorylation in rats, suggesting a mechanism of action for this drug. In addition to this, the expression pattern of cytokines and inflammatory molecules is related to cognitive deficits in SZ patients.

Interestingly, for many of the pathways analyzed, except for the WNT and mTOR pathways which seem to be consistently downregulated in SZ, different studies show variations with respect to the direction of this dysregulation regarding gene or protein expression. This may be a reflection of the high genetic heterogeneity present in SZ, especially in genes described as highly variable for this disease, rather than through gene expression or variant number (Huang et al., 2020; Puvogel et al., 2022). Taking into consideration the variability in gene expression in SZ reviewed in this and other articles, we propose the existence of molecular patterns that may be related to specific symptoms, severity, and even responses to medication. We reviewed several neuro-angiogenic pathways that converge in the neurovascular niche, for which we emphasize the repercussions of their dysregulations expressed as a consistent pattern that may have different impacts on each cell type that is part of the NVU, resulting in sometimes convergent or dissimilar consequences such as biological dysfunction or symptomatology.

There are several noteworthy pathways included in this review that crosstalk and contribute to common functions. For example, NOTCH, WNT, and NF-κB participate in BBB formation and maintenance; HIF, VEGFA, and NOTCH cross-regulate each other in angiogenesis; ROS, WNT, and UPR promote neurogenesis; to mention only a few interactions (Table 1). We have made an effort to dissect the specific contribution of each of these pathways in SZ, in particular, in processes associated with brain angiogenesis and neurodevelopment (Figure 2B). As stated before, for many of the analyzed pathways the main conclusion may be that they are dysregulated in SZ, and whether there is a higher or lower pathway activity that may relate to the progression of the disease or be explained by the use of pharmacological treatment requires further investigation. Interestingly, many of the molecules described in this review can be obtained by peripheral samples, as plasma, which allows measurements of analytes for diagnosis and patient stratification. More effort should be made to deeply understand the specific contribution of each one of these pathways to the SZ onset and progression. miRNAs might be involved in the regulation of multiple cell signaling pathways and may affect cellular physiological functioning, and thus may be involved in the onset of SZ. As stated, recent literature suggest that miRNAs are present in human plasma and could be a potential biomarker of SZ.

In summary, we describe the current state of knowledge regarding neuro-angiogenic pathways and their dysregulation in SZ. As presented, the interpretation of the data reveals the complexity of brain interactions and the SZ physiopathology. We are eager for future research investigating the multicellularity of brain structuration as well as its systemic co-dependence, which, we believe, could be crucial for the emergence of new biomarkers and therapeutic targets in SZ.
